# Some Notes on Stem Cell Therapy in Cardiovascular Diseases

**Published:** 2010

**Authors:** Mohammad Reza Mohammadhasani, Mandana Hasanzad, Amirreza Mohammadhasani, Mohammad Samzadeh, Maryam Eslami

**Affiliations:** 1 Islamic Azad University, Tehran Medical Branch, Tehran, Iran; 2 Research Center of Heart Transplantation and Surgery, Shariati Hospital, Tehran, Iran; 3 Babol University of Medical Sciences, Babol, Iran; 4 Shahid Beheshti University of Medical Sciences, Tehran, Iran

**Keywords:** Stem Cell Therapy, Cardiovascular Disease, Iran

## Abstract

Cardiovascular diseases have become an increasing clinical issue worldwide. Acute ischaemic injury and chronic cardiomyopathies lead to permanent loss of cardiac tissue and ultimately heart failure. Current therapies widely aim to attenuate the pathological changes that occur after injury and to reduce risk factors of cardiovascular diseases. However, they do not improve the patient's quality of life or the prognosis more than moderate. A new challenge in the treatment of the cardiovascular disease is cellular transplantation or cellular cardiomyoplasty. Different types of stem cells have been used for stem cell therapy. Clinical trials using primary bone-marrow-derived cells and skeletal myoblasts have also shown some encouraging results. An additional clinical and pre-clinical study to further enhance the beneficial effects of cell therapy is necessary. Recent studies have shown that there are various pools of putative resident stem cells in an adult heart, raising the hope that these cells can contribute to the treatment of cardiovascular diseases.

## Introduction

Cardiovascular disease is one of the major causes of death worldwide accounting for nearly 17 million deaths per annum according to the World Health Organization Atherosclerosis is a complex trait which arises from the interaction of a number of genetic and environmental factors and complex gene-environment interactions.^1^

**Figure 1:**
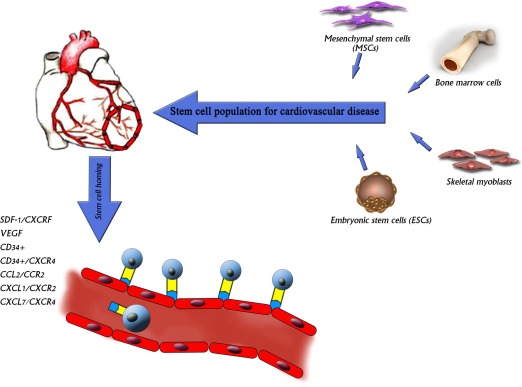
Stem cell therapy for cardiovascular disease. *(Figure design By Arlen Mokhtarzadeh)*

Atherosclerosis involves multiple vascular territories such as carotids, coronaries and peripheral vessels. The molecular mechanisms leading to atherosclerosis are still unclear.^1^ Many epidemiological studies indicated that hypertension, diabetes, obesity, smoking and hyperlipidaemia are risk factors contributing to coronary artery diseases (CAD).^2^–^4^ For those suffering from cardiovascular diseases, stem cell biology represents a new medical frontier. Researchers are working towards using stem cells to replace damaged heart cells and restore cardiac function. Although there is much excitement because researchers nowknow that adult and embryonic stem cells can repair damaged heart tissue, many questions remained to be answered before clinical applications can be made. Unlike other organs such as liver, heart lacks adequate auto-regeneration ability.^5^ There are several therapies for heart failure including medical, surgical and stem cell or progenitor cell therapy. Cell therapy has become a new potential cardiovascular therapeutical tool recently.^6^ The effective goals of cell therapy are myocardial regeneration and neovascularisation. Several different types of stem cells have been studied to find the best source for cardiac regeneration (figure 1).

### Stem cell populations for cardiovascular disease

#### Embryonic stem cells (ESCs)

Embryonic stem cells in mouse and human can be extracted from the inner body of the blastocyst and can be cultured in vitro.^7^, ^8^ From the various stem cell populations studied so far, the ESC cells have the greatest capacity for cardiac cell differentiation and long-term survival.^9^ However, no clinical trial in human for myocardial repair has been applied yet.

#### Skeletal myoblasts

*S*keletal myoblasts which are called satellite cells or skeletal muscle precursors are present in skeletal muscles. These cells are further differentiated than the ESCs. After two or three weeks of cell culture, they can be implanted.^10^ Skeletal myoblasts have the capacity to differentiate in vitro into non-muscle cell types.^11^, ^12^ Finally, there are several barriers that still remain including variability and complexity in the application of skeletal myoblast populations.^13^

#### Bone-marrow-derived stem cells

Bone marrow mononuclear cell can be derived from bone marrow and peripheral blood. Easy isolation, safety and feasibility for their implantation are the advantages to consider, although, investigations found limited or no differentiation of bone marrow cells to cardiovascular cell types.^14^, ^15^ Endothelial progenitor cells (EPCs) is another bone marrow cell type which is important in neovasculogenesis.

#### Mesenchymal stem cells (MSCs)

Mesenchymal stem cells derive from bone marrow and adipose tissue. It is easy to isolate and culture these cells in vitro. The MSCs are multipotent and they are less-immunogenic than others.^16^ A problem with mesenchymal stem cell engraftment was formation of bone and cartilage in heart after transplantation. The microenvironment at the region of engraftment of stem cells is very important and it may cause differentiation of stem cells into unwanted cell types

#### Important parameters in cell therapy

In order to consider cell therapy as clinically relevant, many parameters need to be optimized including cell type, cell function, cell number and route of administration. As mentioned earlier about different cell types for cell therapy, in all controlled studies, significant positive results have been observed in multipotent cells which were transplanted.^17^–^19^ The cell numbers used in cell therapy for chronic coronary artery disease can be between 3.10^6^ to 800.10^6^ cells. There is no certain correlation between the number of cells administered and its effect on heart failure. Another parameter that is associated with cell therapy improvement is the cell functionality.^20^, ^21^ Route of administration which considered in surveys is not a determining factor for successive delivery, although there is no direct comparison between routs of administration in cell therapy for cardiovascular diseases.

#### Mechanism of Cell Therapy

These different stem cell types have different potential mechanisms of action. One of the proposed mechanisms is myogenesis which is more controversial than the other mechanism, particularly in transdifferentiation of bone marrow derived cells into heart cells. It is not yet clear if transplanted cells themselves differentiate or paracrine effects of the transplanted cells stimulate stem cells differentiation or both of these factors act. The other proposed mechanism of cell therapy is angiogenesis. Bone marrow cells may be able to secret multiple angiogenic substances and these cells can be differentiated into cells with the ability to create new blood vessels. Strategies to augment cell function, survival, and homing could be crucial to improve success rates for cell therapy. There are two strategies which may improve the efficiency of cell therapy: 1) pretreatment of the cells *ex vivo* by small molecules or modification of the cells by genes to improve the function, survival, and homing capacity after infusion or injection; and 2) activation of the target tissue to specifically augment signals capable of attracting infused cells or modulating cell function and survival.^22^ Researchers studying the use of embryonic stem cells are also trying to determine why, in animals, most implanted stem cells re-enter the circulation or die rather than engraft to the heart muscle wall to form new muscle cells. They are also searching for the ways to use gene therapy to increase the number of embryonic stem cells that live on as new muscle cells.

#### Stem cell research in Iran

In the Middle East, the highest absolute number of patients with cardiovascular disease is estimated in Iran. While combating major risk factors including hypertension, smoking, and hyperlipidemia, is crucial for improving health of the nation, control programs regarding CAD, via modifying the modifiable risk factors are envisaged.^23^ Several stem cell therapies have been performed in Iran. In a survey in Iran the efficacy of autologous bone marrow derived mesenchymal stem cells in improving heart function in patients with old myocardial infarction was investigated.^24^ In another study in Iran by Ghavamzadeh et al, hematopoietic stem cell therapy was reported to be a choice treatment of many malignant, non-malignant, and genetic diseases. They introduced 105 cellular therapies for post-myocardial infarction, multiple sclerosis, cirrhosis, head of femur necrosis, and renal cell carcinoma. About 30 patients were re-transplanted and about 74.9% of the patients remained alive between one to 168 months after stem cell transplantation. Nearly 25.1% of their patients died after stem cell transplantation. The causes of deaths were relapse, infections, hemorrhagic cystitis, graft versus host disease, and others.^25^

## Discussion

Several phenotypically distinct cell populations have been utilized for heart failure. In summary, pluripotent (e.g., mesenchymal stem cells), totipotent (e.g., embryonic) and multipotent (e.g., tissue specific) stem cells can be applied.^26^ Multipotent stem cells have less ability to differentiate than embryonic stem cells, but embryonic stem cells are able to differentiate into cardiomyocytes.^27^, ^28^ In several studies, it has been shown that several pluripotent stem cells are able to differentiate into cardiomyocytes and lead to neovascularisation such as haematopoietic, mesenchymal and endothelial progenitor stem cells.^29^–^32^ Many researchers now believe that cellular therapy will likely revolutionize approaches to heart failure.

### Future perspectives

Finally, proper cell type, cell number and route of administration still need to be determined and generation of unwanted cell types must be prevented. Researchers have to determine exactly how the stem cells work. The main challenge for developing any stem cell based therapy for heart diseases is the control of cell migration, proliferation and differentiation ex vivo as well as in vivo. The initial trials with patients will be phase I studies. However, the validity for the future of stem cell therapy depends on the benefits obtained in the human phase II and III clinical trials. Stem cell therapy for heart failure needs further investigation and much more work needs to be done. Stem cell therapies in conjunction with current treatments may help improve the quality of life in cardiovascular disease patients.
